# Development of Phosphodiesterase–Protein-Kinase Complexes as Novel Targets for Discovery of Inhibitors with Enhanced Specificity

**DOI:** 10.3390/ijms22105242

**Published:** 2021-05-15

**Authors:** Nikhil K. Tulsian, Valerie Jia-En Sin, Hwee-Ling Koh, Ganesh S. Anand

**Affiliations:** 1 Department of Biological Sciences, 14 Science Drive 4, National University of Singapore, Singapore 117543, Singapore; nikhil.tulsian@nus.edu.sg; 2 Department of Biochemistry, 28 Medical Drive, National University of Singapore, Singapore 117546, Singapore; 3 Department of Pharmacy, 18 Science Drive 4, National University of Singapore, Singapore 117543, Singapore; sin.valerie@u.nus.edu; 4 Department of Chemistry, The Pennsylvania State University, Philadelphia, PA 16801, USA

**Keywords:** phosphodiesterase (PDE), natural products, inhibitors, protein kinase, selectivity, fluorescence polarization

## Abstract

Phosphodiesterases (PDEs) hydrolyze cyclic nucleotides to modulate multiple signaling events in cells. PDEs are recognized to actively associate with cyclic nucleotide receptors (protein kinases, PKs) in larger macromolecular assemblies referred to as signalosomes. Complexation of PDEs with PKs generates an expanded active site that enhances PDE activity. This facilitates signalosome-associated PDEs to preferentially catalyze active hydrolysis of cyclic nucleotides bound to PKs and aid in signal termination. PDEs are important drug targets, and current strategies for inhibitor discovery are based entirely on targeting conserved PDE catalytic domains. This often results in inhibitors with cross-reactivity amongst closely related PDEs and attendant unwanted side effects. Here, our approach targeted PDE–PK complexes as they would occur in signalosomes, thereby offering greater specificity. Our developed fluorescence polarization assay was adapted to identify inhibitors that block cyclic nucleotide pockets in PDE–PK complexes in one mode and disrupt protein-protein interactions between PDEs and PKs in a second mode. We tested this approach with three different systems—cAMP-specific PDE8–PKAR, cGMP-specific PDE5–PKG, and dual-specificity RegA–R_D_ complexes—and ranked inhibitors according to their inhibition potency. Targeting PDE–PK complexes offers biochemical tools for describing the exquisite specificity of cyclic nucleotide signaling networks in cells.

## 1. Introduction

Second-messenger cyclic nucleotides (cNMPs) are important regulators of numerous cellular pathways. Phosphodiesterases (PDEs) catalyze the hydrolysis of cyclic nucleotides regulating the overall levels of cyclic nucleotides and thereby impact the magnitude and duration of the cellular response. This makes them important targets for drug discovery [1,2,3]. The PDE superfamily comprises 11 different families in mammals, each with numerous subtypes and isoforms [4]. Isoforms of various PDEs have been effectively targeted to treat cardiac arrhythmia, inflammation, erectile dysfunction, and steroidogenesis [1]. Based on their substrate specificity, PDEs are categorized broadly into cyclic 3′, 5′ adenosine monophosphate (cAMP)- and cyclic 3′, 5′ guanosine monophosphate (cGMP)- specific and dual-specificity PDEs. About 100 PDEs are thus distributed in various tissues and across different stages of development. Each PDE isoform includes a conserved C-terminal catalytic domain associated with one or more variable N-terminal regulatory domains. Association of PDEs with specific receptors and cyclases to form signaling islands referred to as ‘signalosomes’ has been increasingly recognized to be the primary mode of cyclic nucleotide regulation in cells [5,6,7]. These signaling islands are mediated by specific scaffold proteins that localize multiple elements of the cNMP signaling pathway [8,9,10,11,12,13,14].

In signalosomes, PDEs are anchored in close proximity to cyclic nucleotide receptors and function as multivalent macromolecular assemblies, rather than as free diffusive PDEs [6,15]. An imperative effect of such colocalization of PDEs and cNMP receptors is that the PDEs are poised to hydrolyze cNMP bound to the receptors, i.e. protein kinases (PKs) [16]. This ‘direct’ hydrolysis of bound cNMPs offers precision in regulating various signaling pathways and imparts specificity. Inhibition of PDEs leads to an overall increase in cNMP levels, which is essential in disease control. Due to the ubiquitous presence of PDEs in the body, low selectivity of particular inhibitors to their respective PDEs gets translated into unintended adverse effects in cells. For example, cyanopsia (i.e., blue tinge in vision) is associated with the use of sildenafil, a PDE5-specific inhibitor. This visual symptom occurs due to its cross-reactivity of sildenafil with PDE6, which is present only in rod and cone photoreceptors [17,18]. Hence, this necessitates more isoform-specific inhibitors to overcome the cross-reactivity problem faced by current PDE inhibitors. Targeting the receptor-bound PDE, rather than PDEs alone, overcomes some of the limitations of nonspecific overlapping effects that inhibitors targeting conserved PDE sites might present.

Our goal was to determine the degree of PDE inhibition by different small molecules using PDE–PK complexes as the targets. To screen inhibition of cAMP- or cGMP-specific PDEs, we selectively chose (i) cAMP-specific- PDE8-protein kinase A complex (mammalian), (ii) cGMP-specific- PDE5-protein kinase G (PKG) complex (mammalian), and (iii) a dual cAMP/cGMP selective RegA–PKA system (from *Dictyostelim discoideum*). cAMP-dependent protein kinase A (PKA) and cGMP-dependent protein kinase G (PKG) represent two of the most important cyclic nucleotide effectors [15,19]. Regulatory subunit of PKA (‘PKAR’) and PKG have two non-redundant high-affinity cyclic nucleotide binding (CNB) sites [20,21,22]. PDEs couple to these bound cyclic nucleotides to form PDE–PK complexes with ‘composite active sites’ [6,23]. Given that most intracellular PDEs are localized within signalosomes [8,11,13], the PDE–PK complexes represent relevant high-specificity targets for inhibitor discovery.

In this study, we propose an assay that leverages the reverse modularity inherent in PDE catalysis wherein the catalytic properties of the PDE active site are directly altered by the cNMP receptor (protein kinases) it is bound to. We propose a fluorescence polarization assay for measuring cNMP-specific displacement of fluorescent cAMP/cGMP analogs from PDE–PK complexes. This allows us to screen natural product extracts for potential PDE inhibitory activities and subsequently identify specific inhibitors. Our results reveal that composite active sites of PDE–PK complexes offer an excellent tool for identifying inhibitors with improved specificity for cAMP/cGMP sites and selectivity for single or dual sites. Targeting PDE complexes thereby offers a new route for accelerating the discovery of target receptor-specific PDE inhibitors.

## 2. Results

### 2.1. Designing Competitive cNMP-Dependent Displacement Assay for PDE Inhibitors 

We first set out to assess a phosphodiesterase–protein-kinase complex as a tool to monitor the hydrolysis of cyclic nucleotides and its displacement by small molecules. To monitor real-time association or dissociation of cyclic nucleotides, we used fluorescent analogs of cAMP (2fluo-cAMP, ‘2fc’) and cGMP (2fluo-cGMP, ‘2fg’). Using fluorescence polarization (FP), we first measured the kinetic complexation of catalytic domains of PDEs with their specific protein kinases and tested their stability using cAMP or cGMP as substrates.

#### 2.1.1. Designing a cAMP-Specific Assay Using PDE8–PKAR Complex

**Figure 1:**
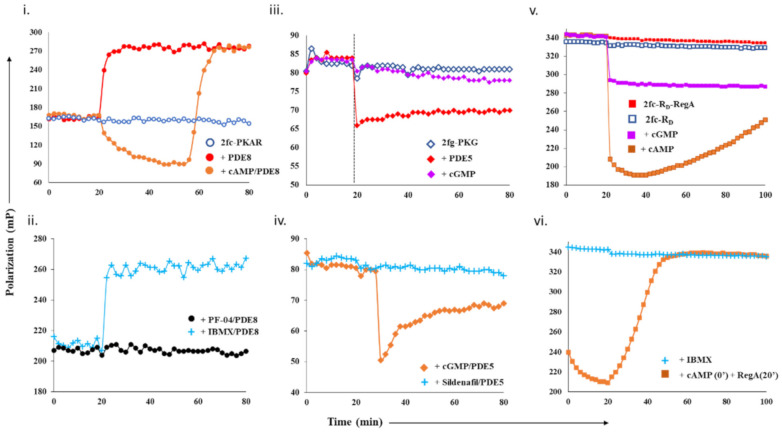
Design and test of competitive displacement assay using PDE–PK complexes. (**i**,**ii**) cAMP-specific probe PDE8–PKAR: (**i**) active PDE8c (red circles) and cAMP-saturated PDE8c (orange circles) were added to 2fc-PKAR at 20 min interval, and the FP values were measured; (**ii**) inhibition and complexation of cAMP-specific PDE8–PKAR were tested by the addition of PDE8c (1 µM) incubated with 10 µM PF-04957325 (black circles) and 500 µM IBMX (cyan plus) to 2fc-PKAR at t = 20 min. (**iii**,**iv**) cGMP-specific probe PDE5–PKG: (**iii**) at 20 min time interval, PDE5 (red diamonds) and cGMP (lilac diamonds) were added to 2fg-PKG (blue circles), and the signal was measured for a total time course of 80 min; (**iv**) PDE5 was incubated with substrate 100 µM cGMP (orange diamonds) and 1 µM sildenafil (cyan plus) and added to 2fg-PKG at time t = 30 min to test stability and inhibition of cGMP-specific PDE5–PKG probe. (**v**,**vi**) Dual-specificity probe RegA-R_D_: (**v**) Plot showing stability of preformed composite active sites of 2fc-R_D_-RegA (red squares) as compared to free 2fc-R_D_ (blue squares). Excess cAMP (orange squares) and cGMP (lilac squares) were added to 2fc-R_D_-RegA complex at 20 min interval and FP measured for further 80 min. (**vi**) At t = 20 min, IBMX (cyan plus) was added to 2fc-R_D_. Separately, cAMP was added to 2fc-R_D_ at time = 0 min, followed by RegA at time = 20 min, to this reaction mixture (orange squares), and FP was measured for a time course of 100 min.

For cAMP-specific PDEs, we used the mammalian PDE8–PKAR system. FP of 2fluo-cAMP-bound PKAR (‘2fc-PKAR’) was constant throughout (Figure 1i, blue), indicating their stable binding. Next, PDE8c in the absence or presence of excess cAMP was added at time t = 20 min. Addition of PDE8c led to an immediate increase in FP values (red plot), suggesting the formation of a complex between PDE8c and 2fc-PKAR, which remained stable over time. Addition of cAMP-saturated PDE8c led to a gradual decrease in FP (orange plot), which indicates competitive displacement of 2fluo-cAMP from PKAR by cAMP. The decrease in FP was followed by a rapid increase in FP, corresponding to those measured for the 2fc-PKAR–PDE8c complex rather than 2fc-PKAR. Such an FP trend suggests that the PDE–PKAR composite active site preferentially hydrolyzed unlabeled cAMP first, followed by reassociation of the 2fc-PKAR–PDE8c complex.

To use the PDE8–PKAR complex as a tool to screen novel inhibitors, we first tested the complex with two known inhibitors—IBMX and PF-04957325. IBMX is a broad-specific PDE inhibitor IBMX, which binds PDE8 but does not inhibit it, while PF-04957325 is a PDE8-specific inhibitor (IC_50_~1–30 nM) [24,25]. PDE8c incubated with IBMX and PF-04957325 was added to 2fc-PKAR to test for inhibition and complexation (Figure 1ii). Consistent with our expectations, we observed that the addition of IBMX-saturated PDE8c (cyan plus plots) resulted in increased FP values similar to those measured for active PDE8c (red plot, Figure 1i). This indicated that IBMX did not inhibit PDE8c, which led to 2fc-PKAR–PDE8c complex formation. Our FP results show no reduction in FP for IBMX-treated PDE8, suggesting that intracellular PDE8 preferred to be associated with cAMP receptors as a composite catalytic site, and therefore preventing PDE8 inhibition by IBMX. This is consistent with literature where IBMX is shown to inhibit other PDEs, but not PDE8, although high-resolution crystal structure showed IBMX-bound PDE8 [26,27]. Addition of PDE8c incubated with PF-04957325 (black circles plot) resulted in no significant change, where FP values remained equivalent to 2fc-PKAR (blue open circles, Figure 1i). Lack of any increase in FP indicated that PF-04957325-inhibited PDE8c was unable to bind to 2fc-PKAR. These results indicate that PDE8–PKAR-composite-site-based fluorescence polarization assay offered a reliable platform to identify inhibitors and distinguish them from small molecules that occupy the catalytic sites.

#### 2.1.2. Designing Assay for cGMP-Specificity Using PDE5–PKG Complex

We next determined the complexation of cGMP-specific PDE5 with PKG and monitored the binding of cGMP and PDE5 to 2fg-PKG (Figure 1iii, blue diamonds). PDE5 addition led to an instant drop in FP (red plot). These results suggest that the PDE5–PKG complex mediated hydrolysis of substrate 2fluo-cGMP, followed by PDE5 dissociation leading to decreased FP. Meanwhile, addition of 1000-fold excess cGMP (lilac plot) resulted in no significant change in FP, indicating that 2fluo-cGMP remained stably bound to PKG. We then set out to determine the effect of excess cGMP on the PDE5–PKG complex. Addition of cGMP-saturated PDE5 (orange diamonds, Figure 1iv) led to a decrease in FP values greater than that observed for active PDE5 alone (red diamonds, Figure 1iii), followed by gradual increased FP equivalent to that of 2fg-PKG–PDE5 mixture. This drop in polarization to lower values indicated cGMP–PDE5 displaced 2fluo-cGMP from PKG, suggestive of processive hydrolysis of cGMP by PDE5–PKG composite site. The hydrolysis product 5′- GMP gets displaced by high-affinity binding 2fluo-cGMP, resulting in increased FP at the end of the reaction.

Subsequently, we tested the substrate displacement from 2fg-PKG by PDE5 in the presence of sildenafil, a PDE5-specific inhibitor [17]. Addition of sildenafil-treated PDE5 did not result in any significant change in FP values, indicative of inhibition of PDE5. These results, therefore, highlight the specificity of using the PDE5–PKG complex as a tool to differentiate between a kinetically active composite site from an inhibited complex.

#### 2.1.3. Designing a Dual cAMP/cGMP Assay Using a Broader Specificity RegA–R_D_ Complex 

As PDE catalytic domains are conserved with high structural similarity, we selected dual-specific cAMP/cGMP selective PDEs. Type 2 phosphodiesterase from *D. discoideum*, RegA, has high similarity to PDE8 catalytic site and therefore was a good choice [28,29]. In addition, RegA is not specific to cAMP and shows cAMP/cGMP selectivity of ~200, indicating it can bind to both cAMP and cGMP. Various studies have shown that RegA regulates the functions of its cognate PKA regulatory subunit (R_D_), which also has two cyclic nucleotide binding sites—CNB:A (high affinity) and CNB:B (low affinity) [19]. Here, our strategy was to differentiate small molecules that displace cAMP and/or cGMP.

We first monitored polarization of the RegA–R_D_ complex (red square) and compared it with FP values of 2fc-R_D_ alone (blue square plot). FP values for the 2fc-R_D_–RegA complex were higher than 2fc-R_D_, indicating stable complex formation (Figure 1v). We then added excess cAMP and cGMP to the 2fc-R_D_–RegA complex to track the association–dissociation changes accompanying substrate hydrolysis. Addition of cAMP (orange squares) resulted in an immediate decrease in FP, indicative of competitive displacement of 2fluo-cAMP by cAMP. Importantly, we observed a gradual and steady increase in FP over the time measured, suggesting slow reassociation of 2fluo-cAMP to R_D_ or the RegA–R_D_ complex. While cAMP addition lead to complete displacement, we noticed that addition of cGMP (lilac plot) resulted in only a partial decrease in FP that remained stable over time. We believe that cGMP displaced 2fluo-cAMP only from one of the two composite sites, probably the low-affinity site of R_D_ and RegA. These results served as controls for displacement from one (like cGMP) or both (like cAMP) composite sites of the dual-specificity PDE–PK complex. Next, we monitored the effects of broad-specificity PDE inhibitor IBMX on the 2fc-R_D_–RegA complex and observed no change in FP (cyan plus plot, Figure 1vi). To simulate intracellular complex formation, cAMP was added to 2fc-R_D_ at 0 min (orange square plot, Figure 1vi), and then RegA at 20 min. Large-scale decrease in FP was observed, followed by increased FP in the presence of RegA. These trends suggested dissociation and reassociation of 2fluo-cAMP from the RegA–R_D_ complex. Here, we developed a competitive displacement assay using the PDE–PK complex as a screening tool to distinguish between cAMP and cGMP specific inhibitors.

### 2.2. Screening Novel Inhibitors in Plant Extracts Using Competitive Displacement Assay

One of the major sources of drugs, especially PDE inhibitors, is natural sources such as plants [30,31,32,33]. Natural products are intrinsically useful in drug discovery due to their high level of structural as well as chemical diversity [34]. They also possess a unique advantage of having high biochemical specificity and binding affinities to their receptors [34]. In this study, the PDE inhibitory potentials of natural products extracted from two medicinal plant species—*Swietenia macrophylla* and *Vitex trifolia*—were investigated using the proposed assay (Supplementary Figure S1). Both these plants have been shown to possess phytochemicals that inhibit PDEs and other enzymes [35]. Profiling of the phytoconstituents in these plant extracts by GC-MS lead to the identification of a wide variety of compounds of differing polarities such as fatty acids, flavonoids, phenolics, methylxanthines, glycosides, and diterpenes (Supplementary Figure S2, Supplementary Table S1). We used the PDE–PK-complex-based fluorescence polarization assay to screen and identify inhibitors. As the fluorescent ligands are the reporters, any competition to their binding to the PDE–PK complex could be monitored easily and rapidly. Further, known PDE inhibitors were used to test the reliability of this assay.

#### 2.2.1. Screening cAMP-Specific Inhibitors Using PDE8–PKAR Complex

**Figure 2:**
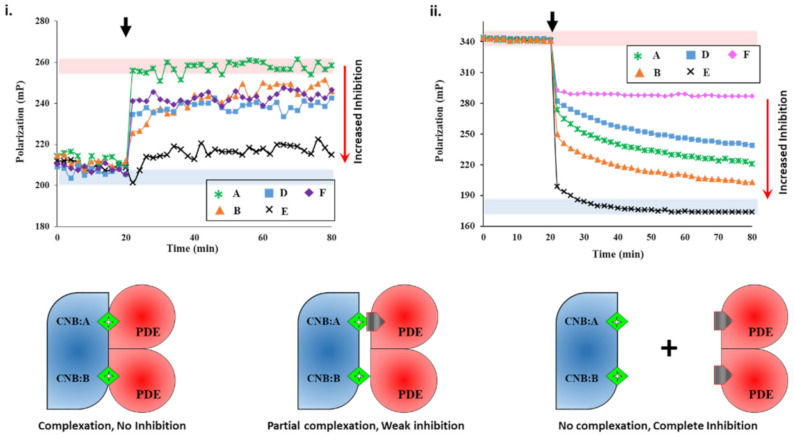
Plant extracts inhibit PDE8 and RegA with different potencies. (**i**) Changes in fluorescence polarization (*y*-axis) over time (*x*-axis) are represented for 2fluo-cAMP-saturated PKAR upon addition of PDE8c pre-incubated with extracts A (green asterisk), B (orange triangles), D (blue squares), E (black cross), and F (lilac diamonds) at 20 min (black arrow). (**ii**) FP plot showing effect of inhibitors on RegA-R_D_ complexation. After 20 min (black arrow), 5 µL of extracts A, B, D, E, and F were added to preformed 2fc-R_D_-RegA complex. Red area indicates zone of no PDE inhibition as per negative control without any extract added, while blue area indicates high PDE inhibition zone using PDE inhibitors as positive control. (C) 2fluo-cAMP (green diamond) is bound to cyclic nucleotide binding (CNB) domains CNB:A and CNB:B of PKA (blue). Cartoons showing inhibitor (gray pentagon) mediated complete (left), partial (center), or no (right) complex formation between PDE (red) and PK (blue) due to no, partial, or complete PDE inhibition, respectively.

**Figure 3:**
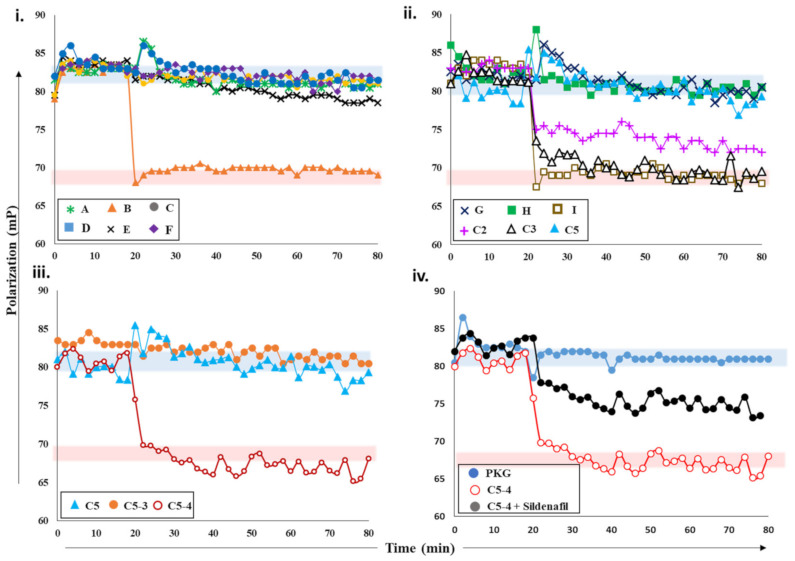
Effects of plant extracts and fractions on PDE5 activity. (**i**) Fluorescence polarization versus time plot depicting the changes in FP values of 2fg-PKG upon addition of PDE5 (0.2 µM) incubated with crude plant extracts A-F at 20 min time interval (black arrow). Red zone indicates values similar to active PDE5 (no inhibition), while blue zone indicates FP values similar to inhibited PDE5. Increased FP indicates greater FP inhibition (red arrow). Inhibition of PDE5 incubated with fractions C2, C3, C5 from crude extract C; sub-fractions C5-3 and C5-4 (panel (**iii**)); and pure compounds G, H, and I (panel (**ii**)). Red area indicates zone of no PDE inhibition based on negative-control active PDE5, while blue area indicates high PDE inhibition zone based on positive-control sildenafil. (**iv**) PDE5 was incubated with sub-fraction C5-4 spiked with 100 nM sildenafil (black circles) and added to 2fg-PKG (blue circles) at time t = 20 min. Sub-fraction C5-4, which does not inhibit PDE5, is shown for reference.

We next set out to screen plant extracts for PDE inhibition and their effects on the PDE–PK complex stability. FP results of IBMX- and PF-04957325 showed the specificity of the assay. To determine if an unknown small molecule was an inhibitor, we used active PDE8 (red area, Figure 2i) and PF-04957325-inhibited PDE8 (blue area, Figure 2i) serving as negative and positive controls for inhibition. First, PDE8 was incubated with 5 µL each of extracts A-F for 15 min and then added to 2fc-PKAR individually at a 20 min interval (Supplementary Table S2). The polarization values observed for extract-A-treated PDE8 (Figure 2i) were similar to those observed for the 2fc-PKAR–PDE8 complex, indicating that the components of extract A were unable to bind or inhibit PDE8 or the PDE8–PKAR composite site. For PDE8 treated with extracts B, D, and E, the FP results observed were in-between active or inhibited composite sites, indicating that these extracts may possess phytoconstituents that block the active site partially (extracts B, D, and E), resulting in a mixture of active and inhibited PDE8–PKAR complexes. Addition of extract-E-treated PDE8 (black cross plot, Figure 3i) resulted in FP values parallel to PF-04957325 (black circles plot, Figure 2i). This suggests that extract E has compounds that bind and block PDE8–PKAR composite site formation and thereby inhibiting its activity. Importantly, these results depict the sensitivity of the assay to screen inhibitors of varying strengths in a single-step procedure. Therefore, the 2fc-PKAR–PDE8 complex can be used as a probe to screen small molecules that may either activate or inhibit phosphodiesterases.

#### 2.2.2. Screening Plant Extracts for Dual cAMP/cGMP PDE Inhibition of the RegA–R_D_ Complex

We next screened the inhibition potency of the plant extracts against cAMP and/or cGMP binding PDEs. At 20 min intervals, the plant extracts were added to RegA–R_D_ complex and their FP was recorded for additional 80 min. Amongst the various plant extracts tested, certain crude extracts showed no change in FP values for the RegA–R_D_ pair (data not shown), while extracts A-F showed significant decreases in polarization values (Figure 2ii). Closer inspection of FP of various extracts highlighted differences in their degree of PDE inhibition. Addition of extract E (black cross, Figure 2ii) to 2fc-R_D_–RegA resulted in a sharp drop in FP, which indicated the rapid displacement of 2fluo-cAMP. Notably, the dissociation kinetics were faster than that observed for cAMP (Figure 1v), suggesting that extract E is highly potent with phytoconstituents that bind to PDE–PK composite site with affinity greater than cAMP. 

Addition of extract A showed a gradual decrease in FP values, although not as potent as extract E, indicating extract A inhibited RegA–R_D_. Interestingly, extract A showed no inhibition of PDE8, which suggests its variability in inhibition of cAMP–PDEs. Addition of extract F (Figure 2ii) led to displacement only from one site, as the FP values were similar to those observed for cGMP (Figure 1v). The potency or the ability of these compounds as potential inhibitors was observed to be E > B > A > D > F in decreasing order, where extract E showed complete and rapid displacement of 2fluo-cAMP, while extract F had the smallest effect. These results highlight the importance of targeting the PDE–PKAR complex as the ‘new active site,’ as it offers insights into the specificity and selectivity of a particular compound.

#### 2.2.3. Screening cGMP-Specific Inhibitors by Targeting PKG–PDE5 Complexes

Next, we screened various plant extracts with a cGMP-specific PDE5–PKG complex. PDE5 treated with sildenafil was used as a positive control to measure PDE5 inhibition (blue area, Figure 3), while active PDE5 (red area) as a control for no inhibition. PDE5 was incubated with various crude plant extracts A-F for 15 min and then added to 2fc-PKG (Figure 3i). Addition of PDE5 treated with extract B (orange triangles) resulted in decreased FP values, similar to active PDE5, while PDE5 treated with other extracts showed no significant decrease in the polarization values and was comparable to sildenafil. These observations suggested that extract B was unable to inhibit PDE5, but other extracts likely consisted of compounds that inhibit PDE5 and the PDE5–PKG composite site. The inhibition potency of these extracts was C = F > A > D > E in decreasing order.

Further characterization of the inhibition of these extracts was carried out using PDE-Glo^TM^ phosphodiesterase assay. The results of FP assay and PDE-Glo^TM^ assay were similar (described in a later section). The two complementary methods allowed us to select active extracts and further downstream processing. Crude extract C was then subjected to fractionation and separation into less complex mixtures. This yielded five fractions C1-5, which were then tested for PDE inhibition (Supplementary Figure S1). PDE5 incubated with extracts C2, C3, and C5 was added to 2fg-PKG and their polarization values recorded (Figure 3ii). Addition of C2-incubated PDE5 led to a decrease in FP values intermediate to those of active and inhibited PDE5, indicating partial/weak inhibition. When C3-incubated PDE5 was added, a greater decrease in FP was observed with values similar to active PDE5, indicative of no PDE inhibition. Fraction C5 (blue triangle) showed the greatest PDE5 inhibition as reflected by the lack of any changes in FP when mixed with 2fg-PKG. 

Subsequently, fraction C5 was subjected to further fractionation to isolate the active compound(s). Fractions C5-3 and C5-4 obtained were then targeted. PDE5 treated with extract C5-4 addition resulted in a decrease in FP (maroon, open circles) with values similar to those of active PDE5. Fraction-C5-3-treated PDE5 addition led to a significant change in FP, with values similar to those observed for 2fg-PKG. These results indicate that sub-fraction C5-4 did not inhibit PDE5, while C5-3 inhibited PDE5 similar to sildenafil. Furthermore, preliminary mass spectrometry analysis (Supplementary Figure S2iiC) of C5-3 fraction led to identification of few components which could possibly be PDE5 inhibitors. We then used commercially available standards G, H, and I of the identified small molecules. We applied our competitive displacement assay using the PDE5–PKG composite site to identify which of these compounds showed inhibition. PDE5 was incubated with three compounds G, H, and I and added to 2fg-PKG. Decreased polarization was observed for compound-I-incubated PDE5, suggesting that this did not inhibit PDE5. FP values for PDE5 treated with compounds G and H did not change over the time course of the experiment, indicating that these two were potential PDE5 inhibitors.

Current methods employed for the screening and detection of PDE5 inhibitors and their analogs as adulterants in herbal products include HPLC-UV, MS-based methods (e.g., LC-MS, GC-MS), vibrational spectroscopic methods (e.g., IR, Raman spectroscopy), and NMR spectroscopy. However, such targeted, structure-based techniques are limited by the need for prior knowledge of the chemical structure and thus have limited use. In contrast, the developed FP assay is an untargeted activity-based bioassay where detection of any adulterants is based on their pharmacological mechanism (i.e., PDE5 inhibition). Thus, its utility as a qualitative screening assay for the detection of PDE5 inhibitor analogs in natural products was investigated. As can be seen from Figure 3iv, addition of PDE5 pretreated with C5-4 alone resulted in a drop in FP values similar to that of active PDE5. This indicates that C5-4 is unable to inhibit PDE5 and is in line with previous results. On the other hand, addition of C5-4 spiked with the sildenafil led to a slight drop in FP values that were between that of active and inactive PDE5. This suggests that the extract spiked with sildenafil partially inhibits PDE5. Therefore, the results indicate that the detection capability of the developed FP assay toward the sildenafil was retained even in a complex matrix such as a semi-purified fraction from a plant extract and may potentially be applied for the screening of herbal products adulterated with PDE5 inhibitors in the future.

PDE5–PKG-composite-site-based assay is capable of rapidly distinguishing between an inhibitor and a non-binding molecule. We believe that this approach has enormous implications in identifying novel compounds that bind to both kinase and phosphodiesterase and can be used as a high-throughput screening procedure.

### 2.3. Ranking Inhibitors Targeting the Specific PDE–PK Complexes

Using various extraction procedures, a range of mixtures were isolated from the two species of medicinal plants examined. Next, we compared the results of our FP assay with a currently available commercial luminescence-based PDE-Glo^TM^ phosphodiesterase assay. In the PDE-Glo^TM^ assay, PDEs were treated with each extract for 15 min, and the activity/inhibition was determined by incubating with cNMP substrate for additional 30 min. The luminescence values were detected according to the manufacturer’s instructions. Extracts from both plants prepared from a wide range of solvents (see methods) showed significant PDE inhibition (*p* < 0.05; compared to control), indicating the presence of multiple active constituents with varying polarities. 

**Figure 4:**
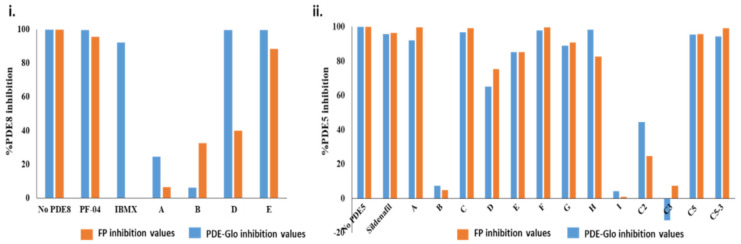
Comparison of PDE-Glo^TM^ and FP assay results for PDE inhibition. The results for fluorescence polarization (orange) and luminescence-based PDE-Glo^TM^ (blue) for PDE8 (**i**) and PDE5 (**ii**) are compared and depicted. No PDE8 or PDE5 was used as a negative control for maximal signal (100%); PF-04957325 (99.8%) and sildenafil (98.5%) inhibitors were used as positive controls for highest inhibition. PDE8 was incubated with extracts A, B, D, and E. Disparity is seen between the two assays for extracts A and B. (**ii**) Effect of extracts A–F, compounds G, H, and I, and fractions from extract C on PDE5 inhibition is compared for the two assays. Inhibition values for FP and PDE-Glo^TM^ assays were calculated as described in methods, and the error bars were too small to be visible. The values are tabulated in Supplementary information Table S3.

PDE-Glo^TM^ phosphodiesterase assay was carried out for extracts A, B, C, D, and E, while sildenafil (for PDE5) and PF-04957325 (for PDE8) were used as positive controls. Results obtained from PDE-Glo™ assay reveal that extracts A, C, and E significantly inhibited PDE5 while extract B did not inhibit PDE5 (Figure 4ii). On the other hand, extracts A and B inhibited PDE8 only moderately, while extracts C and E showed a high degree of inhibition (Figure 4i). The potency of these extracts as potential PDE5 inhibitors in decreasing order was C > A > E, where extract C showed nearly complete inhibition while extract E had the least effects. However, the potency of PDE8 inhibition was observed as E > D > B > A with extract E blocking the PDE8 active site comparable to PF-04957325. Notably, the order of inhibition of PDE5 (Figure 4ii) and PDE8 (Figure 4i) by the plant extracts is different.

PDE-Glo^TM^ assay results for fractions C2 and C3 show low inhibition, while fraction C5 showed high PDE5 inhibition, comparable to that of sildenafil (Figure 4ii). Subsequently, semi-quantitation of the sub-fraction C5-3 and the compounds G, H, and I identified from C5-3 was also carried out. PDE-Glo^TM^ assay results are similar to those observed for FP, with compounds G and H inhibiting PDE5 to ~90%, while PDE5 remained active in the presence of compound I. 

Comparative analysis of luminescence and fluorescence polarization assays revealed PDE-specific inhibition by various extracts (Figure 4, Supplementary Table S3). While the results are consistent for PDE5 with similar % inhibition and extract C being the most potent plant extract for cGMP-specific PDEs, the comparison for PDE8 was different. Incubation of PDE8 with extract B for 15 min showed no inhibition by PDE-Glo^TM^ results (Figure 4i), but the fluorescence polarization results clearly show a time-dependent inhibition (Figure 2i, orange triangles). At earlier times (20–40 min), the FP values were low, indicating PDE8 inhibition, but at later times (60–80 min), the FP values increased to values similar to active PDE8. The PDE-Glo^TM^ phosphodiesterase assay (i) relies on stopping the PDE catalysis using IBMX and (ii) takes ~3 h in total, and thus this assay introduced bias. Thus, extract B showed no inhibition in the luminescence assay, which is similar to FP values observed during later times. Therefore, targeting the PDE–PK composite site by the fluorescence polarization approach provides greater insights into the stability and kinetics of the inhibitor in the catalytic site, as well as reports on the effects of other components in the mixture.

## 3. Discussion

Cyclic nucleotide signaling integrates multiple signaling and metabolic events in cells, and hence its fine-tuned spatio-temporal regulation is critical. With advanced techniques, the PDEs are now recognized to act in conjunction with other macromolecules, notably the cNMP-effector proteins in signalosome assemblies. This increased focus on signalosome assemblies warrants examining PDEs as enzymes complexed with cNMP-target proteins, rather than as free PDEs in solution. Here, in this study, we reported and discussed the importance of targeting the composite PDE–PK active sites for identifying specific inhibitors. We earlier showed that the PDE–PK composite site is catalytically highly efficient and is the preferred route of cyclic nucleotide turnover [14,16] and related signaling events. Through an extensive analysis of different ligands, we proposed the PDE–PK complex as a probe using a time-resolved fluorescence polarization assay that offers a dual readout of binding of small molecules by dissociation/displacement and the degree of enzyme activity or inhibition.

**Figure 5:**
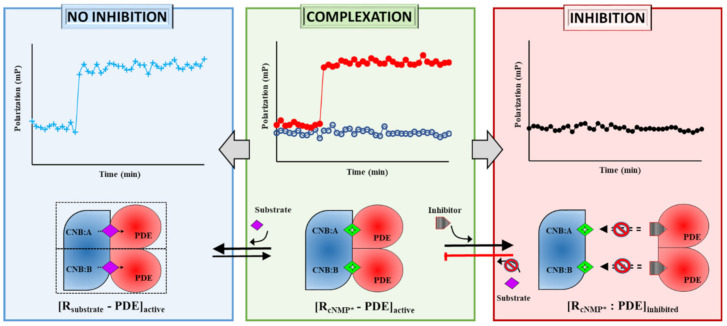
PDE–PK composite site as the new target for drug discovery. (Center panel) The cyclic nucleotide binding domains (CNB) of protein kinase, the ‘receptor’ (R) and catalytic site of phosphodiesterase (‘PDE’, red) coupled in the presence of fluorescent analog of cyclic nucleotide (green diamond, cNMP*) to form an [R_cNMP_*–PDE] active complex (red circles plot). (Left pathway) The stability and activity of the complex were tested by addition of cyclic nucleotide substrates (purple) and resulted in the competitive displacement of the fluorescent analogs and leading to a catalytic active [R_substrate_–PDE] complex. The displaced fluorescent analog of cAMP reassociates with PDE–PK complex resulting in increased FP (cyan plus plot). (Right pathway) Inhibitors (gray) bind to and block the active site and prevent composite site formation yielding an [R_cNMP_–PDE] inhibited complex as observed by no changes in FP (black circles plot).

The phosphate binding sites of protein kinases (PKA/PKG) and the phosphate hydrolysis sites of phosphodiesterases (PDE8/PDE5) couple to form a receptor–hydrolase complex. This coupled active site bound to a fluorescent analog of cNMP*, i.e., the ‘R_cNMP_*–PDE probe’ (Figure 5, green box), serves as an excellent means to determine cAMP/cGMP hydrolysis and their binding kinetics. In earlier studies, using varying concentrations of substrates, we showed enhanced substrate hydrolysis by the PDE–PK composite site followed by reassociation as the R_cNMP_*–PDE probe. PDE hydrolyzes 2fluo-cAMP to 2fluo-AMP, and the PDE–PK complexes bind fluorescent analog of the product as well, offering itself as a robust probe to monitor the active, inactive, and end-state complexes. In this study, we assessed the applicability of three PDE–PK complex systems as a probe for rapid screening of novel inhibitors. Small molecules that activate (Figure 5, blue box), or are weak competitive inhibitors with a delayed response, or show strong enzymatic inhibition (Figure 5, red box) can be successfully screened using this time-resolved FP assay. Besides comparing known and novel inhibitors, semi-quantitative analysis of efficacy (IC_50_) and duration of inhibition can be inferred as well. For instance, extract E displaces 2fluo-cAMP instantly from the RegA–R_D_ complex, faster than cAMP, indicating that the affinity of compounds in extract E is higher than cAMP (<2 nM).

This R_cNMP*_–PDE probe senses the changes in fluorescence polarization intensities measured over the course of the reaction and gives a readout of enzyme kinetics. Fluorescence polarization has been widely applied as a high-throughput screen to identify inhibitors from a large library [36]. Traditionally, PDEs have been targeted alone by fluorescence assays [37] or using radioactive-labeled cyclic nucleotides such as [^3^H], [^14^C], or [^32^P] cAMP/cGMP as substrates [37,38,39] and cell-based assays [40]. Alternatively, luciferase-luciferin coupled detection of ATP levels in cAMP signaling pathway has been developed to quantify the PDE activity by luminescence. However, these assays for screening lead compounds require multiple incubation steps, dose-dependent and PDE-concentration-dependent optimization, and often cell-culture methods. One of the most important concerns of these assays is the non-specificity of the small molecules targeting all PDEs, with little or no information on isoform-specific inhibition. Consequently, this warrants a rapid and robust method to determine the inhibition of specific PDEs. Recently, an NMR-based phosphodiesterase assay was developed based on the quantification of the product 5′-AMP [41]. This method is limited by the requirement of NMR expertise and is based on free PDEs as targets. To this end, our approach of targeting a specific PDE–PK probe allowed the screening of various test molecules rapidly, without any optimization requirements.

Here, we compare the results of the screening of extracts from two species of medicinal plants by a commercial luminescence assay and our proposed fluorescence polarization assay. Although the luminescence provides an overall end-point view of the PDE inhibition by the various extracts, it lacks mechanistic insights into the specificity of PDE inhibition. On the other hand, with the fluorescence polarization assay using the PDE–PK probe as the target, we uncovered more details on the inhibitory effects of these extracts (Table 1).

Firstly, our assay differentiates between extracts that are composed of numerous natural products with varying inhibition potency. While other assays provide an end-point result, our approach gives a time-dependent readout of the effects of inhibitors. For instance, extract B strongly inhibits the PDE8–PKAR complex at early times but does not bind stably, as observed by decreased inhibition at later stages. On the contrary, according to luminescence assay, extract B does not inhibit PDE8 or PDE5. Consequently, limitations of existing PDE assays would have deemed extract B as non-inhibitory, while our FP assay clearly shows that this extract may inhibit PDE. Secondly, using different model systems, we could distinguish broad-specific inhibitors from isoform-specific inhibitors (Table 1). Our results show that extracts A and E are broad-specificity inhibitors, and extract B, compounds G and H are isoform-specific inhibitors. Thirdly, the PDE–PK composite probe allowed us to determine the substrate specificity of the extracts to either cAMP or cGMP. Luminescence assay qualifies it as a PDE inhibitor, but fluorescence polarization assay specifies it as a cGMP-specific inhibitor. Together these results emphasize the importance of targeting PDEs as complex active sites rather than free PDEs.

In literature, free PDEs have been the sole targets for drug design and small molecule therapeutics. In many cases, this approach has led to the discovery of inhibitors with unintended side effects. A majority of approved compounds interact at the conserved catalytic sites and result in cross-reactivity with ‘off-target’ proteins. With over 100 PDEs present, there is, therefore, an urgent need to identify isoform-specific inhibitors [42]. Our proposed assay is based on competitive binding between substrate and novel inhibitors targeting PDE–PK complexes, a representative of intracellular signalosomes. Here, we showed that the PDE–PK complex acts as a sensitive and rapid fluorescence polarization probe and is able to provide insights into lead compounds or identify natural products as potential PDE–PK inhibitors. Development of new bioinformatics algorithms and molecular docking tools have provided opportunities in the screening of potential small molecule inhibitors with insights into their mechanism of action. In the near future, interdisciplinary studies focusing on complementation of virtual docking and screening methods like ours would benefit in a higher proportion of hit-to-lead compounds in drug discovery [43,44].

## 4. Materials and Methods

### 4.1. Materials

Ultra-competent *Escherichia coli* BL-21 (DE3) bacterial strains used for protein expression were obtained from Life Technologies (Carlsbad, CA, USA). TALON^®^ cobalt resin for affinity purification was from Clontech (Mountain View, CA, USA) while BioGel HTP hydroxyapatite beads were from Bio-Rad Laboratories (Hercules, CA, USA). Analytical grade organic solvents (hexane, dichloromethane, ethyl acetate, ethanol, and methanol) and HPLC-grade methanol were purchased from Tedia (Fairfield, OH, USA). Milli-Q (Merck, Darmstadt, Germany) water was used. PDE-Glo™ Phosphodiesterase Assay kit was purchased from Promega Corporation (Madison, WI, USA). LC/MS-grade acetonitrile, methanol, and water were from Fisher Scientific (Waltham, MA, USA), and trifluoroacetic acid (TFA), sequence-analysis-grade, was from Fluka BioChemika (Buchs, Switzerland). Deuterium oxide was from Cambridge Isotope Laboratories (Tewksbury, MA, USA). All other reagents and chemicals were research-grade or higher from Sigma-Aldrich (St. Louis, MO, USA). 

Active recombinant human PDE5A was from SignalChem Pharmaceuticals Inc. (Richmond, Canada), and active recombinant human cGMP-dependent protein kinase type 1 (PKG1) was obtained from Sigma-Aldrich (St. Louis, MO, USA). Dimethyl sulfoxide (DMSO) was procured from Sigma-Aldrich (St. Louis, MO, USA) while phosphate buffer saline (PBS) was from Vivantis Inc. (Oceanside, CA, USA). Sildenafil citrate was acquired from Tokyo Chemical Industry Co. Ltd. (Tokyo, Japan) while 3-isobutyl-1-methylxanthine (IBMX) was from Sigma-Aldrich (St. Louis, MO, USA). PF-04957325 was from MedChemExpress (Singapore). Fluorescent analogs 2’-fluo-AHC-cGMP (2’-(6-[fluoresceinyl] aminohexylcarbamoyl) guanosine-3′, 5′-cyclic monophosphate) and 2’-fluo-AHC-cAMP (2’-(6-[fluoresceinyl] aminohexylcarbamoyl) adenosine-3′, 5′-cyclic monophosphate), henceforth referred to as ‘2fluo-cGMP’ and ‘2fluo-cAMP’, respectively, were obtained from Biolog Life Science Institute (Bremen, Germany). Compound G was obtained from Sigma-Aldrich (St. Louis, MO, USA), and Compound H from Tokyo Chemical Industry (Singapore).

### 4.2. Methods

#### 4.2.1. Expression and Purification of Proteins

Recombinant proteins used in this study were PKAR (regulatory subunit of bovine cAMP-dependent protein kinase PKA), R_D_ (regulatory subunit of *Dictyostelium discodieum* cAMP-dependent protein kinase PKA), PDE8c (catalytic domain of human PDE8A1), and RegA (type 2 phosphodiesterase from *Dictyostelium discodieum*). Each of these proteins was recombinantly expressed in *E. coli* competent cells and purified as described previously [6,16,19]. The purity (>95%) and homogeneity of each of the proteins were confirmed using size-exclusion chromatography and denaturing acrylamide gel electrophoresis. cGMP-dependent protein kinase (PKG) and cGMP-specific phosphodiesterase (PDE5) were obtained commercially.

#### 4.2.2. Preparation of Plant Extracts

Two medicinal plant species [35,45,46] were collected locally, and their voucher specimens were kept at the Herbarium of Department of Pharmacy, National University of Singapore. Extraction using different solvents (e.g., ethanol, methanol, water) and extraction methods (maceration, ultrasonication, Soxhlet extraction) yielded different plant extracts, and extracts A to F were used in this study. Extracts A to C were obtained from *Swietenia macrophylla* King seed kernels (Plant 1) and D-F from *Vitex trifolia* L. var. trifolia leaves (Plant 2) (Supplementary Figure S1). The extracts were subsequently dried in vacuo and separately dissolved in an appropriate volume of 4% *v*/*v* DMSO in PBS to obtain a concentration of 5 mg/mL each.

#### 4.2.3. High-Performance Liquid Chromatography (HPLC)

The crude extracts were subjected to fractionation by HPLC. The extracts were dissolved in HPLC-grade methanol to a concentration of 5 mg/mL and filtered (Filtrex 0.45 μm nylon syringe filter, Agilent, Santa Clara, CA, USA) prior to HPLC analysis. Separation of the crude extract was performed on an Agilent 1260 Infinity HPLC (USA) equipped with diode array detector (DAD) using an Agilent Eclipse XDB-C18 reversed-phase column (4.6 mm × 250 mm, 5 μm). Five microliters of each extract were injected at a flow rate of 1 mL/min, using water and acetonitrile as mobile phases A and B, respectively (Supplementary Figure S2). The solvent gradient used was as follows: 10% B at 0–5 min, 15–50% B at 5–15 min, 50–90% B at 15–40 min, and 90–100% B at 40–55 min. Spectral peaks were detected over wavelength range of 190–400 nm, and UV detection wavelengths were set at 230, 254, and 280 nm.

Normal-phase adsorption chromatography was performed on extract C using silica gel (Si-gel) 60 (0.063–0.200 mm, Merck, Germany). Stepwise elution was performed using n-hexane-ethyl acetate (EA)-methanol (MeOH), starting with n-hexane:EA (1:0–0:1, *v*/*v*), followed by EA:MeOH (1:0–0:9, *v*/*v*) as a gradient elution system. Based on their thin-layer chromatography profiles, similar fractions were pooled together to afford fractions C1 to C6. Fraction C5 was subsequently selected (based on its PDE5 inhibitory activity) for further fractionation to yield sub-fractions C5-1 to C5-4.

#### 4.2.4. Gas Chromatography-Mass Spectrometry (GC-MS)

Plant extracts were dissolved in HPLC-grade methanol to obtain a concentration of 1 mg/mL and injected into an Agilent 7890A GC-MS system (Foster City, CA, USA). A DB-5ms column (30.0 m × 0.25 mm) of 0.25 μm film thickness was used with helium as the carrier gas set at 50 mL/min. The initial oven temperature was set at 80 °C, which was then increased to 160 °C at 20 °C/min and subsequently to 300 °C at 5 °C/min. The final temperature was held for 15 min, making the total run time 47 min. The injection volume was 1 μL using a splitless mode. Mass spectrometer was operated in the electron impact (EI) mode, operated within a 20–500 amu mass range. Compounds were identified by conducting a similarity search using National Institute of Standards and Technology (NIST) Mass Spectral Library versions 27 and 247 (Gaithersburg, MD, USA) and Wiley Mass Spectral Database v.7 (USA).

Fractions and sub-fractions showing highest inhibition were subjected to profiling by gas chromatography-mass spectrometry (GC-MS). Compounds belonging to different chemical classes were identified (Supplementary Figure S2, Supplementary Table S1). Based on the degree of inhibition, three biomolecules identified from extract C were selected and their commercial standards (compounds G, H, and I) were obtained and used for further testing.

For *S. macrophylla*, non-polar and moderately polar extracts seemed to be more active as compared to polar extracts. For example, low % PDE inhibition was observed for the water extracts (B) whereas the other extracts (A, C) that were obtained using less polar solvents such as dichloromethane and ethyl acetate showed % PDE inhibition comparable to sildenafil. On the other hand, extracts obtained from *V. trifolia* using solvents of varying polarities demonstrated significant % PDE5 inhibition. Extract D obtained using water as the extraction solvent, while extracts E and F were obtained in methanol. This suggests the presence of multiple active phytoconstituents with varying polarities in *V. trifolia* that contribute to PDE inhibition.

#### 4.2.5. Fluorescence Polarization

Fluorescence polarization (FP) assays were performed using PDE-hydrolyzable fluorescent analogs 2fluo-cAMP and 2fluo-cGMP. Initially, PKAR, R_D_, and PKG were incubated for 18 h with a molar excess of these fluorescent ligands to saturate their CNB sites. Unbound ligands were removed by dialysis and size-exclusion chromatography. Each of these ligands binds its respective receptors with very high affinity and does not dissociate easily [20,47,48]. These proteins bound to analogs are henceforth referred to as ‘2fc-PKAR’, ‘2fc-R_D_’, and ‘2fg-PKG’ for the respective proteins bound to 2fluo-cAMP or 2fluo-cGMP, respectively. FP assays were performed in 96-well flat-bottom opaque non-binding black plates, and measurements were recorded using Synergy 4 multi-detection microplate reader (BioTek, Winooski, VT, USA). For all ligands, excitation wavelength λex = 485 nm and emission wavelength λem = 524 nm were used with a bandwidth of 20 nm and instrument G-factor being 0.87.

##### Designing the PDE–PK Complex as Target

In the first set of experiments (Supplementary Table S2, Experiment 1), FP of 2fc-PKAR (0.5 µM), 2fg-PKG (0.1 µM), and 2fc-R_D_ (1 µM) were recorded for initial 20 min. Then:

- Free PDE8c (1 µM) and PDE8c saturated with cAMP (250 µM), IBMX (500 µM), or PF-04 (10 µM) were added separately to 2fc-PKAR at t = 20 min.
- To 2fg-PKG, cGMP (100 µM) and PDE5 (0.2 µM) with or without sildenafil (1 µM) and cGMP were added independently, and their FP values were measured for additional 60 min.
- FP values of 2fc-R_D_ (1 µM) and 2fc-R_D_ with RegA (2 µM) were recorded for 20 min, followed by addition of cAMP (250 µM), cGMP (250 µM), or IBMX (500 µM) to preformed 2fc-R_D_–RegA complex, and FP was measured for total time of 100 min.

##### Testing PDE–PK Complex with Known Inhibitors

In the second set of experiments (Supplementary Table S2, Experiment 2), IBMX (500 µM) or PF-04957325 (1 µM) saturated PDE8c was added to 2fc-PKAR at time t = 20 min, and the FP was recorded for further 60 min. PDE5 incubated with cGMP (100 µM) or sildenafil (1 µM) was added to 2fg-PKG after 30 min of initial baseline FP measurements. For RegA–R_D_ pair, two different set-ups were tested. First, cAMP (250 µM) was added to 2fc-R_D_ initially (0 min) followed by adding RegA at 20 min; second, IBMX (250 µM) was added to 2fc-R_D_–RegA complex at 20 min. FP was then measured for total time of 100 min.

##### Targeting PDE–PK Complexes with Plant Extracts

In a third experimental set-up (Supplementary Table S2, Experiment 3), PDE8c (1 µM) was incubated with extracts A-F for 15 min and added to 2fc-PKAR at 20 min. To preformed 2fc-R_D_–RegA complex, 5 µL of extracts A-F (1 mg/mL) were added at 20 min, and the FP was recorded for total time of 80 min.

PDE5A (1 µM) was incubated with 5 µL of plant extracts A-F, C2, C3, C5, C5-3, C5-4, and compounds G, H, and I (1 mg/mL each) for 15 min and added to 2fg-PKG in separate wells, and the FP was measured for additional 60 min. In addition, PDE5 was also incubated with sub-fraction C5-4 and spiked with 100 nM sildenafil for 15 min, and then added to 2fg-PKG. Consecutive FP measurements were taken at time intervals of 2 min. All experiments were carried out in duplicates, and averages from three independent measurements were then plotted.

FP-based inhibition of PDE by the extracts was calculated as normalized ratios of differences in polarization of active and inhibited PDE to differences in polarization of PDE-bound and free protein kinases (PKs), as per the following equation:

$$\%\;\mathrm{PDE}\;\mathrm{inhibition}\;\left(\mathrm{FP}\right)\;=\;\left(1-\;\frac{\mathrm{FP}\left(\mathrm{sample}\right)-\mathrm{FP}\left(\mathrm{PDE}\right)}{\mathrm{FP}\left(\mathrm{PK}\right)-\mathrm{FP}\left(\mathrm{PDE}\right)}\;\right)\;\times \;100\%$$

where FP(PDE) is the average FP value of active PDE bound to PK, FP(sample) is the average FP of PDE incubated with extracts/compounds, and FP(PK) is the average FP value of protein kinase without PDE.

#### 4.2.6. PDE-Glo™ Phosphodiesterase Assay

PDE inhibitory activities of the plant extracts were also investigated using the PDE-Glo™ phosphodiesterase assay as per the manufacturer’s instructions. In brief, 5 μL of plant extract/known inhibitor was incubated at room temperature with 7.5 μL of the active recombinant human PDE5 (0.3 ng/μL) or PDE8c (0.3 ng/μL) for 15 min. The final concentration of plant extracts in each well was 1 mg/mL, while that of sildenafil was 1 μM, IBMX at 100 µM, and PF-04957325 at 1 µM. PDE reaction was then initiated by adding 12.5 μL of 20 μM cGMP or 2 μM cAMP (1 mM for PDE8c) to each reaction and incubating the mixture at room temperature for 1 h. The reactions were then terminated, and the luminescence was detected according to the manufacturer’s instructions.

PDE-Glo^TM^ phosphodiesterase assay measures PDE-mediated catalysis of cyclic nucleotides indirectly by conversion of ATP levels by luciferin–luciferase system. The assays were performed in 96-well opaque white plates from SPL Life Sciences (Busan, Korea), and luminescence was measured using Promega GloMax^®^-Multi Detection System (Madison, WI, USA). The luminescent signal produced is directly proportional to the remaining ATP levels and hence directly correlates with PDE activity. Thus, the greater the inhibition potency of the plant extracts/fractions, the lower the luminescent signal detected. PDE aliquoted in 4% DMSO in PBS was used as negative control, while test well containing only cGMP (i.e., no inhibitor and no PDE) was also included as PDE activity control (akin to 100% PDE inhibition). PDE inhibition of the plant extracts/known inhibitor was calculated using the following equation:

$$\%\;\mathrm{PDE}\;\mathrm{inhibition}\;\left({\mathrm{PDE}}_{\mathrm{Glo}}\right)\;=\;1-\frac{[\mathrm{RLU}\left(\mathrm{sample}\right)]}{\mathrm{RLU}(\mathrm{negative}\;\mathrm{control})}\;\times \;100\%$$

where RLU (sample) is the average relative luminescence units of the specific sample/plant extract, and RLU (negative control) is the average relative luminescence units detected for the negative control. The results were generated from at least three independent experiments and each experiment performed in duplicates.

## 5. Conclusions

Current repertoire of available assays lacks the ability to profile the isoform-specificity, efficacy, and potency of the inhibitors as these are designed to target exclusively free PDEs. Free PDEs passively hydrolyze bulk cyclic nucleotides in the cytoplasm and represent a subset of the total pools of intracellular PDEs. We proposed a single-step, sensitive and reproducible method for rapid screening of inhibitors with greater specificity and lower cross-reactivity. The PDE–PK complex as a pharmacological target offers a new route for accelerating the discovery of a new class of inhibitors for precise regulation of compartmentalized cyclic nucleotide signaling and preventing unwanted cross-talk.

## Figures and Tables

**Table 1:** Summary of the % inhibition of extracts obtained from fluorescence polarization assay results using specific PDE–PK pair as the probe.

	% Inhibition			
Extract	PDE8–PKAR	PDE5–PKG	RegA–R_D_	Characteristics
A	6.5%	99.8%	61.1%	Potent inhibition of cGMP-selective PDEs
B	32.6%	4.9%	75.0%	Partial inhibition of cAMP-specific PDEs
D	40.1%	75.5%	48.6%	Partial inhibition of general PDEs
E	88.6%	85.3%	99.5%	Potent inhibition of general PDEs
F	30%	98%	50%	Inhibition of cGMP-selective PDE sites
Sildenafil	- *	96.5%	n.d *	Known PDE5-specific inhibitor
PF-04957325	95.6%	- *	n.d *	Known PDE8-specific inhibitor

* n.d.: not determined.

## Supplementary Materials

The following are available online at https://www.mdpi.com/article/10.3390/ijms22105242/s1.

## Abbreviations

2fc—2’-fluo-AHC-cAMP; 2fg—2’-fluo-AHC-cGMP; cAMP—cyclic 3′,5′ adenosine monophosphate; cGMP—cyclic 3′,5′ guanosine monophosphate; cNMP—cyclic nucleotide monophosphate; PKAR—regulatory subunit of protein kinase A; R_D_—regulatory subunit of protein kinase A of *Dictyostelium discoideum*; 2fc-PKAR—PKAR bound to 2fc; 2fc-R_D_—R_D_ bound to 2fc; 2fg-PKG—protein kinase G bound to 2fg; PDE—phosphodiesterases; PDE–PK—general phosphodiesterase–protein-kinase complex.

## References

[1] Conti M., Beavo J. (2007). Biochemistry and physiology of cyclic nucleotide phosphodiesterases: Essential components in cyclic nucleotide signaling. Annu. Rev. Biochem..

[2] Jin S.L., Lan L., Zoudilova M., Conti M. (2005). Specific role of phosphodiesterase 4B in lipopolysaccharide-induced signaling in mouse macrophages. J. Immunol..

[3] Tsai L.C., Beavo J.A. (2012). Regulation of adrenal steroidogenesis by the high-affinity phosphodiesterase 8 family. Horm. Metab. Res..

[4] Beavo J.A. (1988). Multiple isozymes of cyclic nucleotide phosphodiesterase. Adv. Second Messenger Phosphoprot. Res..

[5] Dodge K.L., Khouangsathiene S., Kapiloff M.S., Mouton R., Hill E.V., Houslay M.D., Langeberg L.K., Scott J.D. (2001). mAKAP assembles a protein kinase A/PDE4 phosphodiesterase cAMP signaling module. EMBO J..

[6] Krishnamurthy S., Moorthy B.S., Xin Xiang L., Shan L.X., Bharatham K., Tulsian N.K., Mihalek I., Anand G.S. (2014). Active site coupling in PDE:PKA complexes promotes resetting of mammalian cAMP signaling. Biophys. J..

[7] Raymond D.R., Wilson L.S., Carter R.L., Maurice D.H. (2007). Numerous distinct PKA-, or EPAC-based, signalling complexes allow selective phosphodiesterase 3 and phosphodiesterase 4 coordination of cell adhesion. Cell Signal..

[8] Baillie G.S., Scott J.D., Houslay M.D. (2005). Compartmentalisation of phosphodiesterases and protein kinase A: Opposites attract. FEBS Lett..

[9] Soderling S.H., Beavo J.A. (2000). Regulation of cAMP and cGMP signaling: New phosphodiesterases and new functions. Curr. Opin. Cell Biol..

[10] Gold M.G., Gonen T., Scott J.D. (2013). Local cAMP signaling in disease at a glance. J. Cell Sci..

[11] Wong W., Scott J.D. (2004). AKAP signalling complexes: Focal points in space and time. Nat. Rev. Mol. Cell Biol..

[12] Conti M., Mika D., Richter W. (2014). Cyclic AMP compartments and signaling specificity: Role of cyclic nucleotide phosphodiesterases. J. Gen. Physiol..

[13] McCormick K., Baillie G.S. (2014). Compartmentalisation of second messenger signalling pathways. Curr. Opin. Genet. Dev..

[14] Tulsian N.K., Ghode A., Anand G.S. (2020). Adenylate control in cAMP signaling: Implications for adaptation in signalosomes. Biochem. J..

[15] Torres-Quesada O., Mayrhofer J.E., Stefan E. (2017). The many faces of compartmentalized PKA signalosomes. Cell Signal..

[16] Tulsian N.K., Krishnamurthy S., Anand G.S. (2017). Channeling of cAMP in PDE-PKA Complexes Promotes Signal Adaptation. Biophys. J..

[17] Birch D.G., Toler S.M., Swanson W.H., Fish G.E., Laties A.M. (2002). A double-blind placebo-controlled evaluation of the acute effects of sildenafil citrate (Viagra) on visual function in subjects with early-stage age-related macular degeneration. Am. J. Ophthalmol..

[18] Laties A., Zrenner E. (2002). Viagra (sildenafil citrate) and ophthalmology. Prog. Retin. Eye Res..

[19] Krishnamurthy S., Tulsian N.K., Chandramohan A., Anand G.S. (2015). Parallel Allostery by cAMP and PDE Coordinates Activation and Termination Phases in cAMP Signaling. Biophys. J..

[20] Ogreid D., Ekanger R., Suva R.H., Miller J.P., Doskeland S.O. (1989). Comparison of the two classes of binding sites (A and B) of type I and type II cyclic-AMP-dependent protein kinases by using cyclic nucleotide analogs. Eur. J. Biochem..

[21] Kim J.J., Casteel D.E., Huang G., Kwon T.H., Ren R.K., Zwart P., Headd J.J., Brown N.G., Chow D.C., Palzkill T. (2011). Co-crystal structures of PKG Ibeta (92-227) with cGMP and cAMP reveal the molecular details of cyclic-nucleotide binding. PLoS ONE.

[22] Huang G.Y., Kim J.J., Reger A.S., Lorenz R., Moon E.W., Zhao C., Casteel D.E., Bertinetti D., Vanschouwen B., Selvaratnam R. (2014). Structural basis for cyclic-nucleotide selectivity and cGMP-selective activation of PKG I. Structure.

[23] Wilson L.S., Elbatarny H.S., Crawley S.W., Bennett B.M., Maurice D.H. (2008). Compartmentation and compartment-specific regulation of PDE5 by protein kinase G allows selective cGMP-mediated regulation of platelet functions. Proc. Natl. Acad. Sci. USA.

[24] Tsai L.C., Shimizu-Albergine M., Beavo J.A. (2011). The high-affinity cAMP-specific phosphodiesterase 8B controls steroidogenesis in the mouse adrenal gland. Mol. Pharmacol..

[25] Vang A.G., Basole C., Dong H., Nguyen R.K., Housley W., Guernsey L., Adami A.J., Thrall R.S., Clark R.B., Epstein P.M. (2016). Differential Expression and Function of PDE8 and PDE4 in Effector T cells: Implications for PDE8 as a Drug Target in Inflammation. Front. Pharmacol..

[26] Soderling S.H., Bayuga S.J., Beavo J.A. (1998). Cloning and characterization of a cAMP-specific cyclic nucleotide phosphodiesterase. Proc. Natl. Acad. Sci. USA.

[27] Wang H., Yan Z., Yang S., Cai J., Robinson H., Ke H. (2008). Kinetic and structural studies of phosphodiesterase-8A and implication on the inhibitor selectivity. Biochemistry.

[28] Moorthy B.S., Gao Y., Anand G.S. (2011). Phosphodiesterases catalyze hydrolysis of cAMP-bound to regulatory subunit of protein kinase A and mediate signal termination. Mol. Cell Proteom..

[29] Shaulsky G., Fuller D., Loomis W.F. (1998). A cAMP-phosphodiesterase controls PKA-dependent differentiation. Development.

[30] Abusnina A., Lugnier C. (2017). Therapeutic potentials of natural compounds acting on cyclic nucleotide phosphodiesterase families. Cell Signal..

[31] Kumar A., Sharma V., Singh V.P., Kaundal M., Gupta M.K., Bariwal J., Deshmukh R. (2015). Herbs to curb cyclic nucleotide phosphodiesterase and their potential role in Alzheimer’s disease. Mech. Ageing Dev..

[32] Bischoff E. (2004). Potency, selectivity, and consequences of nonselectivity of PDE inhibition. Int. J. Impot. Res..

[33] Rahimi R., Ghiasi S., Azimi H., Fakhari S., Abdollahi M. (2010). A review of the herbal phosphodiesterase inhibitors; future perspective of new drugs. Cytokine.

[34] Molinari G. (2009). Natural products in drug discovery: Present status and perspectives. Adv. Exp. Med. Biol..

[35] Sin V.J., Anand G.S., Koh H.L. (2020). Botanical Medicine and Natural Products Used for Erectile Dysfunction. Sex. Med. Rev..

[36] Huang W., Zhang Y., Sportsman J.R. (2002). A fluorescence polarization assay for cyclic nucleotide phosphodiesterases. J. Biomol. Screen.

[37] Dell’Agli M., Galli G.V., Dal Cero E., Belluti F., Matera R., Zironi E., Pagliuca G., Bosisio E. (2008). Potent inhibition of human phosphodiesterase-5 by icariin derivatives. J. Nat. Prod..

[38] Temkitthawon P., Hinds T.R., Beavo J.A., Viyoch J., Suwanborirux K., Pongamornkul W., Sawasdee P., Ingkaninan K. (2011). Kaempferia parviflora, a plant used in traditional medicine to enhance sexual performance contains large amounts of low affinity PDE5 inhibitors. J. Ethnopharmacol..

[39] Ko W.C., Shih C.M., Lai Y.H., Chen J.H., Huang H.L. (2004). Inhibitory effects of flavonoids on phosphodiesterase isozymes from guinea pig and their structure-activity relationships. Biochem. Pharmacol..

[40] Titus S.A., Li X., Southall N., Lu J., Inglese J., Brasch M., Austin C.P., Zheng W. (2008). A cell-based PDE4 assay in 1536-well plate format for high-throughput screening. J. Biomol. Screen.

[41] Akimoto M., Yu T., Moleschi K., Van K., Anand G.S., Melacini G. (2020). An NMR based phosphodiesterase assay. Chem. Commun. (Camb.).

[42] Maurice D.H., Ke H., Ahmad F., Wang Y., Chung J., Manganiello V.C. (2014). Advances in targeting cyclic nucleotide phosphodiesterases. Nat. Rev. Drug Discov..

[43] Pinzi L., Rastelli G. (2019). Molecular Docking: Shifting Paradigms in Drug Discovery. Int. J. Mol. Sci..

[44] Shubina V., Niinivehmas S., Pentikainen O.T. (2015). Reliability of Virtual Screening Methods in Prediction of PDE4B-inhibitor Activity. Curr. Drug Discov. Technol..

[45] Siew Y.Y., Zareisedehizadeh S., Seetoh W.G., Neo S.Y., Tan C.H., Koh H.L. (2014). Ethnobotanical survey of usage of fresh medicinal plants in Singapore. J. Ethnopharmacol..

[46] Sin V.J.-E. (2020). Medicinal plants and cyclic nucleotide phosphodiesterase inhibitory activity. Ph.D. Thesis.

[47] Koutalos Y., Brown R.L., Karpen J.W., Yau K.W. (1995). Diffusion coefficient of the cyclic GMP analog 8-(fluoresceinyl)thioguanosine 3′,5′ cyclic monophosphate in the salamander rod outer segment. Biophys. J..

[48] Schafer P.H., Parton A., Gandhi A.K., Capone L., Adams M., Wu L., Bartlett J.B., Loveland M.A., Gilhar A., Cheung Y.F. (2010). Apremilast, a cAMP phosphodiesterase-4 inhibitor, demonstrates anti-inflammatory activity in vitro and in a model of psoriasis. Br. J. Pharmacol..

