# Supplementary material for: Development of Phosphodiesterase–Protein-Kinase Complexes as Novel Targets for Discovery of Inhibitors with Enhanced Specificity
Source: Int J Mol Sci. 2021 May 15;22(10):5242. doi: 10.3390/ijms22105242 (PMC8156604; doi:10.3390/ijms22105242)
Supplement: Supplementary file 1 [file ijms-22-05242-s001.zip › ijms-1191812-supplementary.pdf]

International Journal of *Molecular Sciences*

MDPI

Article

Development of Phosphodiesterase-Protein Kinase Complexes as Novel Targets for Discovery of Inhibitors with Enhanced Specificity

Nikhil K. Tulsian[1,2], Valerie Jia-En Sin[3], Hwee-Ling Koh[3*], Ganesh S. Anand[1,4*]

[1] Department of Biological Sciences, 14 Science Drive 4, National University of Singapore, Singapore – 117543
[2] Department of Biochemistry, 28 Medical Drive, National University of Singapore, Singapore – 117546
[3] Department of Pharmacy, 18 Science Drive 4, National University of Singapore, Singapore – 117543
[4] Department of Chemistry, The Pennsylvania State University, Philadelphia, United States of America - 16801
* Correspondence: KHL: phakohhl@nus.edu.sg; Tel.: +6565167962; GSA: gsa5089@psu.edu; Tel.: 814-867-1944

SUPPLEMENTARY INFORMATION

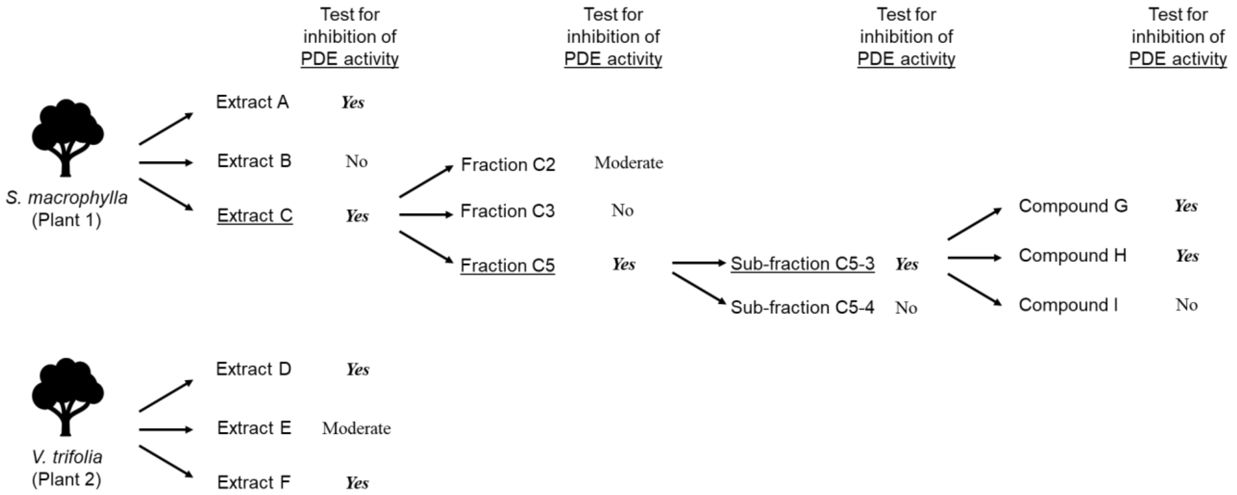

Supplementary Figure S1: Activity-guided fractionation of plant extracts.
Schematic of extraction of compounds/fractions from two species of medicinal plants - *Swietenia macrophylla* and *Vitex trifolia*. From each plant, three crude extracts were isolated and their PDE inhibition was tested by PDE-Glo™ and FP assays. Each of the active/inhibitory fractions were then subjected to another round of fractionation and activity measurement. Qualitative results of activity assay are shown and the extracts/fractions selected for sub-fractionation are underlined.

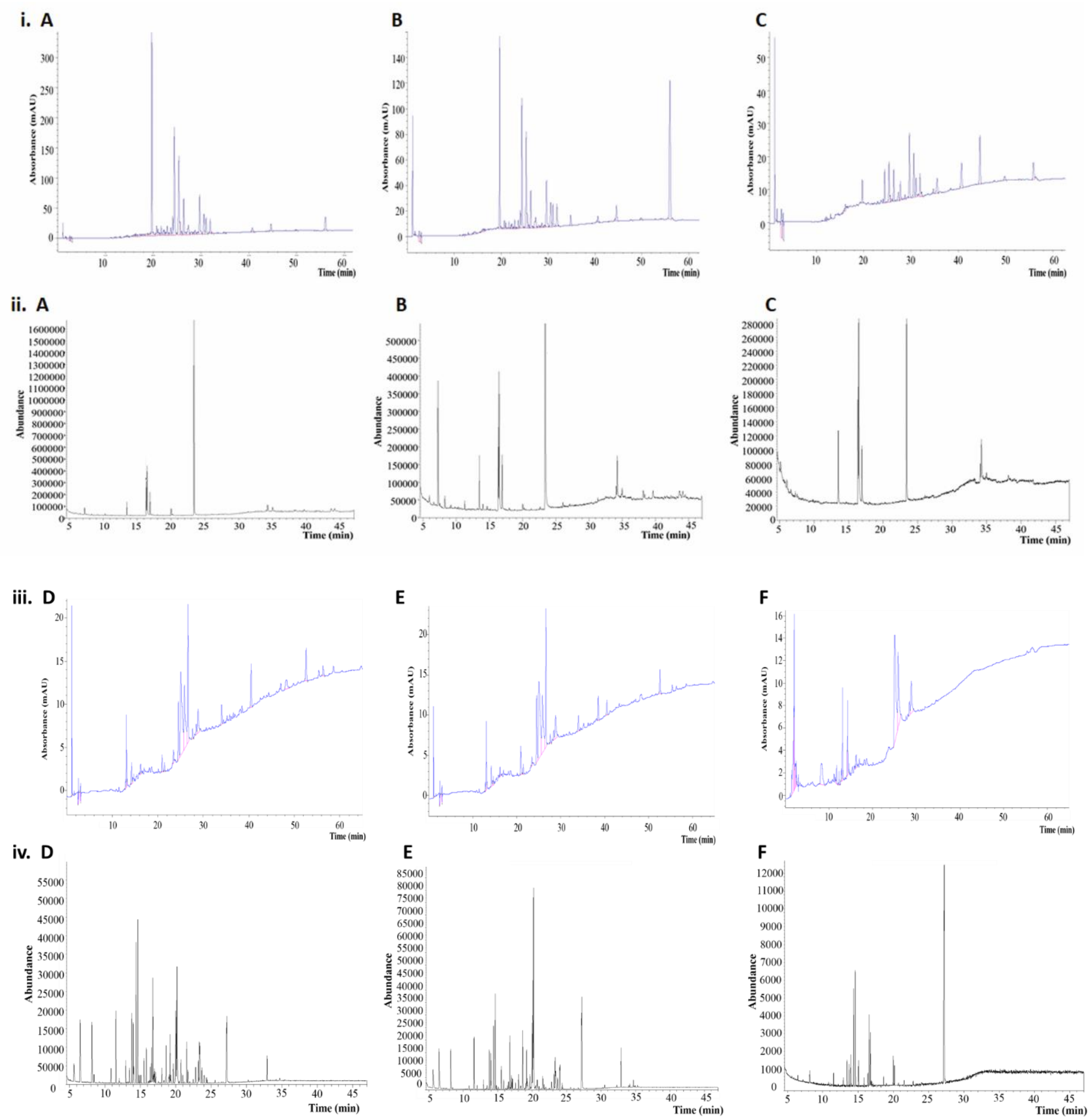

Supplementary Figure S2: Determining chemical composition of the crude extracts by HPLC and GC-MS. Representative ultraviolet absorbance traces (blue curves) determined by HPLC (i, iii) and their corresponding total-ion chromatograms (black curves) detected by GC-MS (ii, iv) are shown for extracts from (i, ii) *S. macrophylla* (extracts A-C) and (iii, iv) *V. trifolia* (extracts D-F). Peak fractions from HPLC were collected and then subjected to GC-MS as described in methods and Supplementary Table S1.

Supplementary Table S1: Summary of chemical composition determined by HPLC and GC-MS profiles of the six crude extracts dissolved in specific solvents.

Plant	Extract	Solvent	Classes of chemical compounds obtained
S. macrophylla	A	Ethanol, Water	Phenolics, methyl esters, lipids
	B	Water	Limonoids, Fatty acids
	C	Ethyl Acetate	Fatty acids and derivatives, Flavonoids
V. trifolia	D	Methanol, Water	Alkyl compounds, Terpenes,
	E	Ethanol, Water	Fatty acids, lignans, amino acid derivatives
	F	Methanol, Water	Esters of fatty acids, Glycosides

Supplementary Table S2: Summary of fluorescence polarization experiments performed for PDE8-PKAR, PDE5-PKG and RegA-R_D systems, indicating the details of components added to the reaction mixtures at various time intervals.

**Experiment 1: Designing the PDE-PK complex as target (related to Figure 1)**			
	**Initial components at 0 min**	**Component added at 20 min**	**Purpose**
**PDE8-PKA**	2fc-PKAR	PDE8c	Complexation
		PDE8c + cAMP	Substrate (cAMP) hydrolysis
**PDE5-PKG**	2fg-PKG	cGMP	Competition with substrate
		PDE5	Complexation
**RegA-RD**	2fc-RD	buffer	Control
	2fc-RD-RegA	cAMP	Displacement by high-affinity substrate
		cGMP	Displacement by low-affinity substrate

**Experiment 2: Testing PDE-PK complex with known PDE-inhibitors (related to Figure 1)**			
	**Initial components at 0 min**	**Component added at 20 min**	**Purpose**
**PDE8-PKA**	2fc-PKAR	PDE8c + IBMX	Non-specific inhibitor
		PDE8c + PF-049573425	Inhibition by specific inhibitor
**PDE5-PKG**	2fg-PKG	PDE5 + Sildenafil	Inhibition by specific inhibitor
		PDE5 + cGMP	Substrate (cGMP) hydrolysis
**RegA-RD**	2fc-RD-RegA	IBMX	Non-specific inhibitor

	2fc-RD + cAMP	RegA	Substrate hydrolysis

**Experiment 3: Testing PDE-PK complex with plant extracts (related to Figure 2)**			
	**Initial components at 0 min**	**Component added at 20 min**	**Purpose**
**PDE8-PKA**	2fc-PKAR	PDE8c + extract A	Screening natural extracts for inhibition of cAMP-specific PDE
		PDE8c + extract B	
		PDE8c + extract D	
		PDE8c + extract E	
		PDE8c + extract F	
**RegA-RD**	2fc-RD-RegA	extract A	Screening natural extracts for inhibition of dual-specificity PDE-PK complex
		extract B	
		extract D	
		extract E	
		extract F	

**Experiment 3: Testing PDE-PK complex with plant extracts (related to Figure 3)**			
	**Initial components at 0 min**	**Component added at 20 min**	**Purpose**
**PDE5-PKG**	2fg-PKG	PDE5 + extract A	Screening natural extracts for inhibition of cGMP-specific PDE
		PDE5 + extract B	
		PDE5 + extract C	
		PDE5 + extract D	
		PDE5 + extract E	
		PDE5 + extract F	
	2fg-PKG	PDE5 + fraction C2	Testing PDE inhibition by isolated fractions
		PDE5 + fraction C3	
		PDE5 + fraction C5	
		PDE5 + compound G	Testing PDE inhibition by compounds identified from fractions
		PDE5 + compound H	
		PDE5 + compound I	
	2fg-PKG	PDE5 + sub-fraction C5-3	Testing PDE inhibition by sub-fractions
		PDE5 + sub-fraction C5-4	
	2fg-PKG	PDE5 + sildenafil + subfraction C5-4	Comparing PDE inhibition by sub-fraction spiked with known inhibitor

Supplementary Table S3: Inhibition values for extracts A-F, fractions C2-C5, and compounds G-I inhibiting PDE8 and PDE5, as determined by fluorescence polarization (FP) and PDE-Glo™ assays. Average values (n = 6) were calculated as described in methods and their standard deviations (SD) are indicated.

**(i) PDE8**					
**S.No.**	**Sample**	**%FP inhibition**	**SD**	**%PDE-Glo inhibition**	**SD**
1	No PDE8	100	0.15	99.95	0
2	PF-04	95.6	2.10	99.6	0.65
3	IBMX	0.24	1.96	92.3	6.4
4	A	6.56	0.55	24.7	6.1
5	B	32.58	2.90	6.4	9.6
6	D	40.2	1.05	99.7	2.3
7	E	88.56	2.78	99.71	2.60

**(ii) PDE5**					
**S.No.**	**Sample**	**%FP inhibition**	**SD**	**%PDE-Glo inhibition**	**SD**
1	No PDE5	100.00	0.10	100.00	0.00
2	Sildenafil	96.50	1.20	95.90	2.30
3	A	99.83	2.50	92.20	6.40
4	B	4.96	0.35	7.40	6.10
5	C	99.10	3.10	96.80	1.95
6	D	75.50	2.20	65.10	9.60
7	E	85.37	1.62	85.30	2.30
8	F	99.83	3.58	97.90	0.65
9	G	90.90	2.60	89.12	5.76
10	H	82.60	1.30	98.53	1.26
11	I	1.00	0.07	4.27	8.15
12	C2	24.80	1.37	44.67	8.54
13	C3	7.40	0.13	-12.70	3.70
14	C5	95.80	2.80	95.65	1.30
15	C5-3	99.10	3.20	94.40	1.74
